# Diversity in *E. coli* (p)ppGpp Levels and Its Consequences

**DOI:** 10.3389/fmicb.2020.01759

**Published:** 2020-08-12

**Authors:** Beny Spira, Katia Ospino

**Affiliations:** Department of Microbiology, Institute of Biomedical Sciences, University of São Paulo, São Paulo, Brazil

**Keywords:** (p)ppGpp, polymorphism, growth rate, evolution, stress resistance, antibiotic resistance, virulence

## Abstract

(p)ppGpp is at the core of global bacterial regulation as it controls growth, the most important aspect of life. It would therefore be expected that at least across a species the intrinsic (basal) levels of (p)ppGpp would be reasonably constant. On the other hand, the historical contingency driven by the selective pressures on bacterial populations vary widely resulting in broad genetic polymorphism. Given that (p)ppGpp controls the expression of many genes including those involved in the bacterial response to environmental challenges, it is not surprising that the intrinsic levels of (p)ppGpp would also vary considerably. In fact, null mutations or less severe genetic polymorphisms in genes associated with (p)ppGpp synthesis and hydrolysis are common. Such variation can be observed in laboratory strains, in natural isolates as well as in evolution experiments. High (p)ppGpp levels result in low growth rate and high tolerance to environmental stresses. Other aspects such as virulence and antimicrobial resistance are also influenced by the intrinsic levels of (p)ppGpp. A case in point is the production of Shiga toxin by certain *E. coli* strains which is inversely correlated to (p)ppGpp basal level. Conversely, (p)ppGpp concentration is positively correlated to increased tolerance to different antibiotics such as β-lactams, vancomycin, and others. Here we review the variations in intrinsic (p)ppGpp levels and its consequences across the *E. coli* species.

## Diversity of (p)ppGpp Concentrations–Impact on Growth Rate and Beyond

“The study of bacterial growth is the essence of microbiology” (Jacques Monod).

The success of an organism in evolutionary terms resides in its ability to reproduce and perpetuate its genes. It would thus be expected that bacterial resources would be devoted most of the time to maximizing growth rate. This may be true under some circumstances, especially, under optimal laboratory growth conditions. However, bacteria actually keep growth rate under very tight control. At the core of growth regulation is a small nucleotide that appears in two different forms—guanosine tetra- and penta-phosphate—ppGpp and pppGpp, collectively known as (p)ppGpp. The grip of (p)ppGpp on growth rate is achieved mainly through an effective inhibition of stable RNA (rRNA and tRNA) synthesis during amino acid starvation and other nutritional stresses in a process that became known as the stringent control (Cashel and Gallant, 1968; Potrykus and Cashel, 2008; Potrykus et al., 2011). Nevertheless, the impact of (p)ppGpp on cell physiology goes far beyond stable RNA control. (p)ppGpp also inhibits DNA replication, lipid and protein synthesis and ultimately cell division (Potrykus and Cashel, 2008; Traxler et al., 2008). Whenever the growth conditions deteriorate, (p)ppGpp concentration increases, severely repressing the expression of growth-related genes. This repression is necessary in order to promote the reallocation of resources, which are then shifted from growth promotion to the maintenance of amino acid as well as energy pools and to cell protection and survival. In fact, (p)ppGpp concentration increases stepwise according to the severity of nutrient depletion (Traxler et al., 2011).

**Figure 1:**
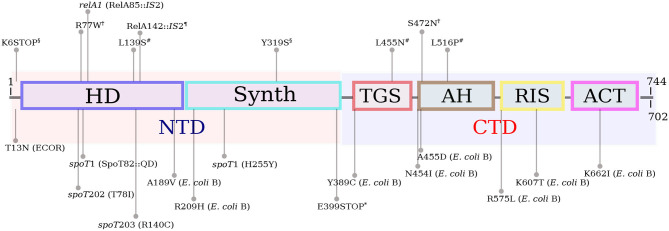
Schematic representation of the long RSH (RelA/SpoT) architecture, as per Atkinson et al. (2011) and Loveland et al. (2016). The RelA and SpoT proteins contain 744 and 702 amino acids, respectively. The NTD half of RSH proteins harbors the catalytic HD (hydrolytic) and Synth (Synthetic) domains. In RelA, the HD domain is not active. The CTD portion of the protein contains four regulatory domains: TGS (ThrRS, GTPase, SpoT/RelA domain), AH (α-helical domain), RIS (Ribosome-InterSubunit domain) and ACT (Aspartate kinase-Chorismate mutase-TyrA domain). The mutations in RelA and SpoT mentioned in the main text are shown above and below the protein diagram, respectively. *relA*1, *spoT*1, *spoT*202 and *spoT*203 are known mutations present in many *E. coli* K-12 derivatives. The *spoT*1 allele consists of two mutations. All mutations, with the exception of those that are followed by *E. coli* B or ECOR (in parentheses) were found in K-12 strains. The T13N substitution is common in strains of the ECOR collection (Ferenci et al., 2011). The SpoT mutations in *E. coli* B were selected in an evolution experiment in glucose-limited minimal medium (Cooper et al., 2003). Mutations ending with “*” were selected during adaptation to high temperature (Kishimoto et al., 2010); “¶” labels indicate RelA mutations selected for n-butanol tolerance (Reyes et al., 2012); “#” labels point to mutations in RelA selected under high ethanol concentration (Horinouchi et al., 2015); RelA mutations ending with “†” were selected for isopropanol tolerance (Horinouchi et al., 2017) and “§” labels indicate RelA mutations selected under growth with lactate (Conrad et al., 2009).

In *E. coli* and related bacterial species, (p)ppGpp is synthesized by two different proteins—RelA and SpoT. These proteins evolved by duplication from a bifunctional ancestral RelA/SpoT Homolog (RSH) possessing both (p)ppGpp synthetic and hydrolytic capabilities, resulting in two proteins with overlapping functionalities (Mittenhuber, 2001; Atkinson et al., 2011). The RelA and SpoT proteins contain 744 and 702 amino acids, respectively. Both proteins can be divided in two parts of similar size (Figure 1). The NTD half of the protein harbors the catalytic HD (hydrolytic) and Synth (Synthetic) domains. In RelA, the HD domain is not active. The CTD portion of the protein contains four regulatory domains: TGS (ThrRS, GTPase, SpoT/RelA domain), AH (α-helical domain), RIS (Ribosome-InterSubunit domain) and ACT (Aspartate kinase-Chorismate mutase-TyrA domain) (Atkinson et al., 2011; Loveland et al., 2016). RelA responds to intracellular amino acid imbalancies, such as amino acid starvation, by synthesizing large amounts of (p)ppGpp (Cashel, 1969). RelA carries an inactive (p)ppGpp-hydrolytic domain and does not hydrolyze the alarmone under any conditions. SpoT is a bifunctional enzyme that contains functional (p)ppGpp-synthetic and hydrolytic domains, but displays weak (p)ppGpp-synthetic activity and strong ppGpp hydrolytic activity. The *relA* knockout accumulates ppGpp in response to several environmental stresses, such as carbon and nitrogen (Edlin and Donini, 1971), phosphate (Spira et al., 1995), iron (Vinella et al., 2005), and fatty acid (Battesti and Bouveret, 2006) starvation.

Early in (p)ppGpp research different spontaneous alleles of *relA* and *spoT* have been isolated. For instance, the *spoT*1 allele (Laffler and Gallant, 1974), that confers a spotless phenotype (absence of pppGpp under amino acid starvation), was isolated from the old 58-161 strain and is now common in many K-12 derivatives (Alföldi et al., 1962). Bacteria that carry the *spoT*1 allele overproduce (p)ppGpp both under nutrient starvation and under normal growth conditions. The *spoT*1 allele contains two different mutations - a H255Y substitution in the synthetase domain (Synth) and a two-amino acid insertion between residues 82 and 83 (+QD) in the hydrolytic domain (HD), both at the NTD portion of SpoT (Figure 1). The two amino acid insertions in the HD domain are likely to negatively affect the ppGpp-hydrolytic activity of SpoT resulting in high (p)ppGpp basal levels, while the H255Y substitution hits a conserved residue (Atkinson et al., 2011), but its effect on the (p)ppGpp-synthetic activity of SpoT is hard to predict. Interestingly, *spoT*1 is usually accompanied in many strains by the defective *relA*1 allele, consisting of an IS2 insertion in the HD domain that is likely to disrupt RelA (p)ppGpp-synthetic activity (Metzger et al., 1989). The *relA*1 mutant displays lower ppGpp basal level than the *relA*^+^ strain (Lagosky and Chang, 1980) and does not accumulate (p)ppGpp in response to amino acid starvation. Apparently, the high ppGpp basal level caused by the *spoT*1 allele is compensated by the defect in (p)ppGpp synthesis caused by the presence of *relA*1. It is therefore no wonder that both alleles often appear together in the same genome.

Later on, other *spoT* alleles, such as *spoT*201, *spoT*202 an *spoT*203 were isolated by selection on amino-triazole plates (Sarubbi et al., 1988). Amino-triazole is a herbicide that inhibits the synthesis of histidine. Bacteria that synthesize high levels of (p)ppGpp overcome histidine starvation by inducing the expression of the *his* operon (Rudd et al., 1985). A critical difference between *spoT*201 and the other three alleles was that the former confers an almost normal growth rate. The other alleles (*spoT*202-203) considerably reduced growth rate and for that reason could be transferred only to a *relA*1 background, but not to a bacterium that carries a wild-type *relA* allele. The *spoT* alleles *spoT*202 and *spoT*203 consist, respectively, of T78I and R140C substitutions, both in the HD domain (Potrykus et al., 2011). The molecular nature of the *spoT*201 mutation has not been published. Given the high (p)ppGpp level in strains bearing these alleles, the *spoT*201-203 mutations have probably compromised the ppGppase activity of SpoT.

It became evident that an inverse linear correlation exists between the intrinsic level of (p)ppGpp in a bacterium (basal level under unrestricted growth conditions) and the bacterial growth rate (Sarubbi et al., 1988). This negative correlation was confirmed when *spoT* mutant alleles were transferred to other genetic backgrounds (Spira et al., 2008). The recombinant strains displayed all the hallmarks of the previously analyzed *spoT* mutations, namely slower growth rate, high levels of the sigma factor RpoS (coordinator of the general stress response) and high resistance to environmental stresses (see below).

The above mentioned *relA* and *spoT* alleles and most data on (p)ppGpp physiology and homeostasis were obtained by studying laboratory strains derived from the ancestral K-12 strain. To date very few attempts have been made to analyze (p)ppGpp homeostasis in natural isolates of *E. coli*. In two of these studies, the basal level and starvation-induced levels of (p)ppGpp were assessed in a set of strains derived from the ECOR collection (Ferenci et al., 2011) and in a collection of Shiga toxin-producing *E. coli* (STEC) strains (Stella et al., 2017). The ECOR collection contains 72 strains from various locations and environments and from five phylogenetic groups (A, B1, B2, D, and E) that supposedly represents the variability in the *E. coli* species (Ochman and Selander, 1984). Most ECOR isolates are commensal, but some are pathogenic. The levels of (p)ppGpp in non-limited minimal medium, in response to amino acid starvation or carbon starvation were reported for 33 strains of the ECOR collection. ppGpp concentrations in the ECOR strains treated with serine hydroxamate, an inhibitor of seryl-tRNA synthetase that induces amino acid starvation, were quite similar in all tested strains. However, (p)ppGpp response to carbon starvation was less homogeneous, consistent with the variation in SpoT observed in those strains. A T13N amino acid substitution was common in strains that showed low (p)ppGpp accumulation in response to carbon starvation and was absent in strains presenting high levels of ppGpp (Ferenci et al., 2011). These data suggested that *spoT* is being subjected to microevolutionary pressures.

**Figure 2:**
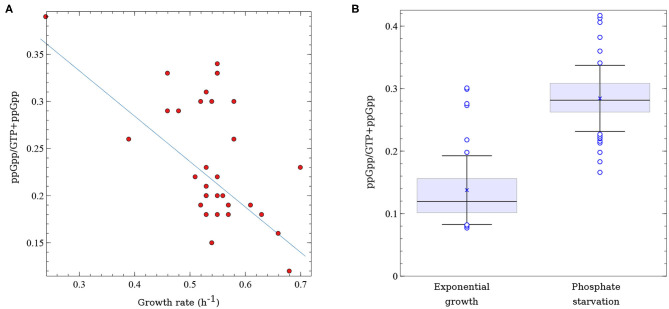
Variability in (p)ppGpp levels across *E. coli* natural isolates. **(A)** Correlation between (p)ppGpp basal levels and growth rate (*h*^−1^) in 33 strains of the ECOR collection growing exponentially in glucose minimal medium. ppGpp levels correspond to the ratio between ppGpp and GTP + ppGpp. Data was extracted from Ferenci et al. (2011). **(B)** Distribution of ppGpp levels (ppGpp/GTP+ppGpp) in 50 STEC isolates growing exponentially in non-limited (glucose) minimal medium or under phosphate starvation. x corresponds to the mean values, horizontal lines represent the median and the error bars correspond to 1 standard deviation; ∘ represent outliers. Data was extracted from Stella et al. (2017).

It is well-established that the intrinsic concentration of (p)ppGpp is inversely correlated with growth rate (Ryals et al., 1982; Sarubbi et al., 1988; Potrykus et al., 2011; Jin et al., 2012). In fact, gratuitous induction of (p)ppGpp synthesis mediated by *relA* overexpression causes an almost instantaneous growth arrest (Schreiber et al., 1991; Svitil et al., 1993; Cruvinel et al., 2019). However, the vast majority of studies that analyzed this correlation used isogenic *E. coli* laboratory strains harboring different *relA* or *spoT* alleles. If growth rate is mainly regulated by (p)ppGpp a good correlation between (p)ppGpp levels and growth rate, even in a set of non-isogenic strains, would be expected. Indeed, when the intrinsic ppGpp concentrations of the ECOR isolates growing under non-limited growth conditions are plotted against their respective growth rates, an inverse correlation is observed (Figure 2A), with a Pearson's correlation coefficient = −0.58. Though not perfect, the inverse correlation between ppGpp concentration and growth rate in these strains validates the central role of (p)ppGpp in governing growth rates across the *E. coli* species, even in strains that come from very different genetic backgrounds as is the case of the ECOR collection. It is worth mentioning that in this as well as in other studies that analyzed (p)ppGpp in exponentially growing bacteria or in response to stresses other than amino acid starvation, pppGpp was below the detection level (Varik et al., 2017; Cruvinel et al., 2019).

In another analysis of (p)ppGpp fluctuation in natural isolates, ppGpp concentration was measured in 50 STEC strains growing under two different culture conditions—non-limited growth medium and phosphate starvation (Stella et al., 2017). A significant variability in ppGpp levels was observed among the STEC isolates (Figure 2B). On average, ppGpp values were twice as high in bacteria submitted to Pi starvation than in the same bacteria growing exponentially in minimal medium. ppGpp values in this set of strains went from 0.08 to 0.30 units for bacteria growing exponentially and from 0.17 to 0.42 units for phosphate-starved bacteria (units correspond to the ratio of ppGpp over GTP+ppGpp). Though this study did not evaluate the variability of ppGpp with growth rate, it correlated the levels of this alarmone with STEC cytotoxicity, as described below.

Altogether, the data presented here highlight the existence of variability in intrinsic ppGpp concentrations across the *E. coli* species and that this variability has a substantial impact on growth rate. However, in addition to growth rate control (p)ppGpp directly and indirectly affects many important bacterial characteristics, such as stress responses, virulence, antibiotic resistance and persistence, biofilm formation, genome stability, and more (Potrykus and Cashel, 2008; Dalebroux et al., 2010; Martin-Rodriguez and Romling, 2017; Rasouly et al., 2017; Hobbs and Boraston, 2019). Variability in (p)ppGpp basal levels is thus likely to affect these traits as well.

It is important to notice that in the studies mentioned above that compared (p)ppGpp values in isogenic and non-isogenic strains, (p)ppGpp was assessed using the classical method of formic acid extraction of ^32^P-labeled bacterial nucleotide pools. These studies did not provide absolute values of (p)ppGpp concentration, but instead presented the level of ppGpp relative to that of GTP+ppGpp as detailed in Cashel (1994). The most relevant limitations of this method is the lack of absolute numerical estimates of (p)ppGpp concentrations and that it leaves out GDP, which constitutes 7.7–15% of the total pool of guanosine nucleotides (Varik et al., 2017), as the resolution of the ^32^P-labeled nucleotides on the TLC plate is not usually good enough to identify GDP spots on the autoradiogram. Because of these limitations, the ppGpp values obtained in those studies cannot be easily compared to the ones found in other reports. However, the relative values of (p)ppGpp obtained by the classical method are reproducible and give a reasonable estimate of (p)ppGpp status in a particular set of strains. More recent techniques for evaluating (p)ppGpp, based on Ion Chromatography-High-Resolution MS (Patacq et al., 2018), HPLC (Varik et al., 2017), or UPLC (Ihara et al., 2015) largely overcome the disadvantages of the ^32^P-classical method.

## Role of (p)ppGpp in Stress Resistance and Nutritional Competence

(p)ppGpp supports survival by either directly or indirectly stimulating the expression of genes involved in stress protection. The cell response to environmental stresses such as extreme pH and osmolarity, dehydration or oxidative stress is coordinated by the sigma factor RpoS (Landini et al., 2014; Schellhorn, 2014), whose synthesis and stability is enhanced by (p)ppGpp (Gentry et al., 1993; Battesti et al., 2011). The culture history of a bacterial population determines its overall physiology, and more specifically, the strength of its response to environmental challenges (Ryall et al., 2012). The specific hurdles that a bacterial lineage experiences throughout its existence would eventually leave their imprints in its genome. For instance, alleles that maintain high levels of RpoS and other stress-related genes would be selected in a population that is being often exposed to environmental stresses Conversely, bacteria growing in a stress-free environment accumulates mutations in genes that downregulate RpoS synthesis, promotes its proteolysis or even acquire null mutations in the *rpoS* gene itself (King et al., 2004; Spira and Ferenci, 2008; Wang et al., 2010). Likewise, genes involved in (p)ppGpp metabolism are under selective pressures driven by culture conditions (Spira et al., 2008; Ferenci et al., 2011). ppGpp pleiotropy indicates that variations in intrinsic (p)ppGpp levels might have broad consequences on bacterial physiology and genotypic characteristics of bacterial populations. Bacteria that display intrinsic high levels of (p)ppGpp are more resistant to environmental stresses either because they express high levels of RpoS or because (p)ppGpp directly stimulates the transcription of other genes related to stress protection. However, the correlation between (p)ppGpp and RpoS is not as straightforward as would be expected from extrapolating data on K-12 strains (Gentry et al., 1993; Spira et al., 2008; Battesti et al., 2011). Analysis of *E. coli* natural isolates does not give a simple relationship in which RpoS concentration is proportional to (p)ppGpp concentration. While some strains exhibit a proportionality between the two measured entities, others display mediocre levels of RpoS but high (p)ppGpp levels (Ferenci et al., 2011). Surely, there are other inputs, other than (p)ppGpp that modulate the levels of RpoS.

**Figure 3:**
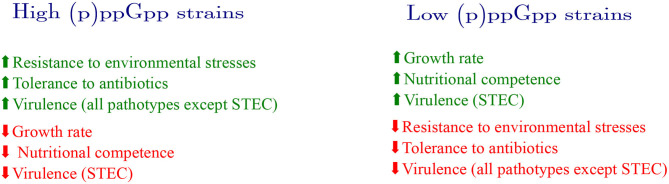
Diversity in (p)ppGpp levels and its consequences. (p)ppGpp intrinsic concentrations are not constant across *E. coli* strains. *E. coli* isolates displaying high (p)ppGpp are benefited under environmental challenges. For instance, these bacteria tolerate higher concentrations of antibiotics and display a higher survival rate when challenged with environmental stresses. Most high-(p)ppGpp pathotypes also show increased ability to colonize host tissues (higher virulence). On the other hand, these strains display lower growth rates and less nutritional competence (ability to utilize alternative carbon sources) than other strains with intrinsically lower (p)ppGpp values. Strains displaying high (p)ppGpp usually carry mutations in the HD domain of SpoT. Mutations in the Synth domain of both RelA or SpoT or in the regulatory domains often cause a reduction in (p)ppGpp levels. The resulting strains display an increased growth rate and higher nutritional competence, but are more sensitive to environmental stresses and antibiotics. STEC is the only known *E. coli* pathotype whose virulence capability benefits from low (p)ppGpp levels.

Both (p)ppGpp and RpoS directly affect the transcription of dozens of genes and indirectly the transcription of many others (Peano et al., 2015; Wong et al., 2017). RpoS competes with other sigma factors, particularly with σ^70^ for binding to the core RNA polymerase. The outcome of this competition is that under nutrient limitation or in the stationary phase (circumstances that cause the accumulation of RpoS), the transcription of σ^70^-dependent genes, i.e., the majority of bacterial genes, is considerably diminished. Hence, the stimulatory effect of (p)ppGpp on RpoS adds another layer of growth control in addition to the already discussed inhibition of stable RNA. Bacterial strains that accumulate high levels of (p)ppGpp or RpoS are less fit for growing on poor carbon sources or under nutrient limitation (King et al., 2004). A trade-off is thus characterized in which a certain bacterial strain cannot simultaneously be nutritionally competent and highly stress resistant (Ferenci, 2016). Figure 3 shows how bacteria with high or low intrinsic (p)ppGpp concentrations deal with environmental challenges.

## Intrinsic (p)ppGpp Concentration as a Target in Evolution Experiments

Given that (p)ppGpp is the most important source of growth rate control (Potrykus et al., 2011), polymorphism in *relA* and *spoT* are likely to occur throughout the course of bacterial evolution and adaptation to different environments, especially in those limited in one or more nutrients, a situation that suppresses normal growth. Several evolution experiments, which resulted in the emergence of mutants related to (p)ppGpp both in batch and in continuous cultures, have been conducted to date. The mutations observed in these studies are summarized in Figure 1.

In one of them, 12 *E. coli* populations have been daily diluted in glucose limited minimal medium and grown for 20,000 generations. Different non-synonymous mutations in *spoT* have been observed in 8 out of 12 evolved populations (Cooper et al., 2003). The first one, A189V is located at the very end of the HD domain; R209H is at the ~45-residues region between the HD and Synth domains; Y389C is at the regulatory TGS domain; N454I and A455D are located at the beginning of the α-helical domain; the mutations R575L and R607L are in the RIS domain and K662I is at the ACT domain. Although (p)ppGpp levels were not measured in this study, the expression of aminoacyl-tRNA synthetases and ribosomal proteins were shown to be upregulated in one of these *spoT* mutants (K662I), suggesting that the mutation caused a reduction in (p)ppGpp intrinsic concentration that led to an increase in growth rate. The ACT domain interacts with the ribosome A site in order to activate the (p)ppGpp synthetic activity (Loveland et al., 2016), thus the K662I substitution is likely to interfere with Synth activation resulting in low (p)ppGpp. A non-sense mutation in the TGS domain of *spoT* (E399*) was observed in another case of adaptive evolution of *E. coli* growing at 43.2°(Kishimoto et al., 2010). This mutant displayed high growth rate at the high temperature, possibly due to a reduction in intrinsic (p)ppGpp levels. This finding is puzzling, once it has been shown that the truncation of the CTD leads to an upshift in (p)ppGpp synthesis (Mechold et al., 2002; Battesti and Bouveret, 2006). However, this particular evolved strain carried additional mutations in *lrp* and *rho* that might have strengthened the observed phenotype.

In another experiment, *E. coli* subjected to adaptive evolution under high ethanol concentrations acquired different mutations in *relA* (L139S, L455N, and L519P) that contributed to an increased tolerance in the presence of 5% ethanol (Horinouchi et al., 2015). According to these authors, the *relA* mutations enabled a relaxed response to ethanol, by diminishing (p)ppGpp concentration, thereby increasing growth rate. The L139S mutation occurred in the pseudo-hydrolytic domain of RelA and is therefore unlikely to affect (p)ppGpp synthesis. The other two mutations—L455N and L516P, were in the TGS and AH domains, respectively. These mutations might have affected the regulation of (p)ppGpp synthesis by RelA as both TGS and AH subunits form the elbow of the boomerang-shaped RelA that interacts with the 30S ribosome and with the deacyl-tRNA (Loveland et al., 2016). A similar study with bacteria growing with increasing concentrations of isopropanol (up to 450 mM) for 210 generations showed that the evolved isolates acquired mutations in *relA* (Horinouchi et al., 2017). Again, the suggested mechanism was that the *relA* mutants expressed RelA proteins that synthesized reduced levels of (p)ppGpp in response to isopropanol, resulting in higher growth rates. The mutations—R77W and S472N, were, as before, in the pseudo-HD and AH domains, respectively.

In another experiment of guided evolution, bacteria grown in a chemostat with increasing butanol concentrations (up to 1.3%) for 144 generations acquired mutations in several genes (Reyes et al., 2012). One of the evolved isolates presented an IS2 insertion at the end of the HD domain of RelA, which has probably compromised the integrity of the entire protein, resulting in a RelA-negative phenotype. Mutations in *relA* also appeared in 2 out of 11 populations growing in lactate minimal medium (Conrad et al., 2009). One mutation—K6*, caused a frameshift at the very beginning of the gene, while the other mutation, Y319S, occurred in the Synth domain of RelA.

In addition to the direct effect of (p)ppGpp on growth, low concentrations of this alarmone also results in reduced levels of RpoS (Gentry et al., 1993; Battesti et al., 2011). Due to the competition between σ^S^ and σ^70^, the former negatively affects the expression of growth-related genes, especially those involved in the uptake and assimilation of alternative carbon sources with a consequent reduction in growth rate (Gentry et al., 1993; King et al., 2004; Magnusson et al., 2005; Spira et al., 2008; Ferenci et al., 2011). Thus, mutations in *relA* would also improve growth by diminishing RpoS concentration in the cell. Figure 3 summarizes the outcomes of bacterial evolution experiments in which mutations in (p)ppGpp-related genes have been observed.

In conclusion, selection of different *relA* and *spoT* alleles in evolution experiments is not uncommon. In fact, in most of these experiments regulatory genes are the primary targets of adaptive selection (Maharjan et al., 2006; Wang et al., 2010). Given the central role that (p)ppGpp plays in the regulation of gene transcription, protein synthesis and growth, it is not surprising that modulation of (p)ppGpp is a primary target for evolution.

## Variability in (p)ppGpp Levels and Its Influence on Antibiotic Susceptibility

The stringent response has been linked to bacterial tolerance to β-lactam antibiotics in *E. coli*. Tolerance to antibiotics is defined as the ability of microorganisms to survive transient exposure to high concentrations of an antibiotic without a change in the minimum inhibitory concentration (MIC) (Brauner et al., 2016). When both the wild-typestrain and *relA* null mutants were exposed to penicillin under amino acid starvation, only the former was able to avoid cell lysis triggered by the presence of the antibiotic (Goodell and Tomasz, 1980; Kusser and Ishiguro, 1985). Moreover, the protective effect of the stringent response against β-lactam antibiotics was reverted by the addition of chloramphenicol (Kusser and Ishiguro, 1985), a well-known inhibitor of the stringent response (Cortay and Cozzone, 1983). In the aforementioned studies (p)ppGpp levels were not directly measured, however, it has been subsequently shown that mecillinam-tolerant mutants accumulated more (p)ppGpp than mecillinam-sensitive strains (Vinella et al., 1992). It became thus evident that high concentrations of (p)ppGpp increase the level of mecillinam tolerance (Joseleau-Petit et al., 1994). The mechanism by which (p)ppGpp confers tolerance to β-lactams was not entirely elucidated. One possibility is that (p)ppGpp acts by inhibiting the biosynthesis of phospholipids. In fact, treatment with cerulenin, an inhibitor of fatty acid biosynthesis, induced β-lactam resistance in the Δ*relA* mutant (Rodionov et al., 1995). In addition, the gratuitous induction of (p)ppGpp accumulation by overexpression of *relA* resulted in the inhibition of phospholipid and peptidoglycan synthesis and in penicillin tolerance (Rodionov and Ishiguro, 1995) supporting the idea that (p)ppGpp mediates penicillin tolerance through the inhibition of phospholipid synthesis (Rodionov and Ishiguro, 1996). However, a more recent study has demonstrated that antibiotic tolerance to β-lactams occurs even in the absence of RelA (Kudrin et al., 2017). *E. coli* cells treated with mupirocin, an isoleucyl-tRNA syntethase inhibitor, displayed increased ampicillin tolerance in the wild-type but not in the relaxed strain. In contrast, the combination of trimethoprim with mupirocin, tetracycline or chloramphenicol significantly increased tolerance to ampicillin in both strains. These data indicate that growth arrest/protein synthesis inhibition can, at least in some cases, increase bacterial tolerance to antibiotics in a (p)ppGpp-independent fashion.

The positive relation between antibiotic tolerance and intrinsic (p)ppGpp concentrations is not restricted to β-lactam antibiotics. The wild-type strain of *E. coli* displayed higher MIC values for trimethoprim, gentamicin and polymixin when compared to the Δ*relA* or Δ*relA* Δ*spoT* mutants (Greenway and England, 1999). The increase in MIC values characterizes an augment in bacterial resistance to these antibiotics (Brauner et al., 2016). Likewise, it has been shown that mutations in the aminoacyl-tRNA synthetase genes *leuS* and *aspS* reduced susceptibility to ciprofloxacin, chloramphenicol, rifampicin, mecillinam, ampicillin, and trimethoprim. Deletion of the *relA* gene in these mutants restored the original MIC values of these antibiotics (Garoff et al., 2018). In another instance bacteria expressing high levels of (p)ppGpp displayed resistance to microcin J25, while strains unable to produce (p)ppGpp were completely sensitive to this antibiotic. In addition, overexpression of *relA* in a strain naturally susceptible to microcin J25 resulted in high MIC values and higher survival rates in killing curves (Pomares et al., 2008).

Several studies have shown a positive correlation between the expression of *hipA*, that encodes a serine/threonine-protein kinase that belongs to a type-II toxin/anti-toxin module, (p)ppGpp production and the formation of persisters (Korch et al., 2003; Bokinsky et al., 2013; Germain et al., 2013; Kaspy et al., 2013). Persistence is the ability of a subpopulation of an antibiotic-sensitive strain to survive for longer periods of time in the presence of high concentrations of an antibiotic than the majority of the population (Brauner et al., 2016). Some strains are able to form a higher percentage of persisters than others. For instance, strains carrying the *hipA*7 allele formed 100-fold more persistent cells than the wild-type strain when exposed to ampicillin. In the absence of (p)ppGpp (Δ*relA* Δ*spoT* double mutant) the *hipA*7 allele did not confer any advantage regarding antibiotic persistence, suggesting that the high-persistence phenotype elicited by *hipA*7 is (p)ppGpp-dependent. Accordingly, overexpression of *relA* in the *hipA*7 strain increased the frequency of persisters (Korch et al., 2003). On the other hand, overexpression of *hipA* granted resistance to ampicillin, but only in *relA*^+^ bacteria, as bacteria overexpressing *hipA* but lacking *relA* were considerably more sensitive to ampicillin. Interestingly, the level of (p)ppGpp in the *relA*^+^ strain overexpressing *hipA* was as high as under amino acid starvation (Bokinsky et al., 2013). Two other studies confirmed the findings of Bokinsky et al. and extended their observations to fluoroquinolone antibiotics (Germain et al., 2013; Kaspy et al., 2013). In addition, these studies suggested a mechanism for *hipA* stimulation of persistence via (p)ppGpp. In their model *hipA* inactivates the glutamyl-tRNA synthetase GltX resulting in the accumulation of uncharged tRNAs which ultimately leads to the activation of RelA and (p)ppGpp synthesis.

Formation of persister cells in bacteria exposed to ofloxacin and ampicillin was also observed upon carbon source transitions, a situation that causes the accumulation of (p)ppGpp (Amato et al., 2013; Amato and Brynildsen, 2015). Deletion of *relA* abolished the formation of ampicillin, but not of ofloxacin persistence, which required the deletion of both *relA* and *spoT*. Furthermore, by controlling the level of (p)ppGpp it has been shown that formation of ampicillin persisters required higher concentrations of (p)ppGpp than formation of ofloxacin persisters. It has also been shown that under conditions of nitrogen starvation *E. coli* accumulates high levels of (p)ppGpp and forms high percentages of persisters when treated with ciprofloxacin, but only in a *relA*^+^ strain (Brown, 2019).

Integrons are important elements in the dissemination of antibiotic resistance genes. It has been shown that (p)ppGpp plays a role in the regulation of *intI*1, which encodes an integrase protein found in class 1 integrons (Strugeon et al., 2016). Accumulation of (p)ppGpp causes the stalling of RNA-polymerase and the formation of R-loops, which in turn activates the SOS response. The autoproteolysis of the *intI*1 repressor, LexA, ensues resulting in the transcription of *intI*1. *In trans* expression of this gene in the Δ*relA* Δ*spoT* double mutant resulted in reduced *intI*1 promoter activity when compared to the parental strains. Overall, these data indicate that (p)ppGpp helps propagating antibiotic resistance genes through activation of integrase in class 1 integrons.

## Variability in (p)ppGpp–Effect on Bacterial Pathogenicity

The expression of virulence-related genes in pathogenic *E. coli* is very well-integrated with (p)ppGpp homeostasis Dalebroux et al. (2010). For instance, (p)ppGpp influences the ability of enterohemorrhagic *E. coli* (EHEC) to colonize the host intestine (Nakanishi et al., 2006). This *E. coli* pathotype secretes a potent cytotoxin—Shiga toxin, that causes serious diseases in humans—bloody diarrhea and HUS (hemolytic uremic syndrome). In addition, bacteria of this pathotype harbor a 35 Kb pathogenicity island known as the Locus of Enterocyte Effacement (LEE), which carries most genes implicated in EHEC intimate adherence (Nguyen and Sperandio, 2012). The passage from the nutrient-rich higher intestine to the nutrient-limited lower intestine triggers the accumulation of (p)ppGpp, which in turn stimulates the transcription of the LEE operons. The EHEC Δ*relA* mutant was unable to induce bacterial adherence or expression of the LEE (Nakanishi et al., 2006). Overexpression of *relA* greatly stimulated the expression of EspB and Tir, two proteins encoded by the LEE and increased the transcription of several LEE genes, implying a positive correlation between (p)ppGpp concentration and EHEC virulence. EPEC (Enteropathogenic *E. coli*) is another diarrheogenic pathotype that carries the LEE, but unlike EHEC it does not produce Shiga toxin. EPEC strains harbor a plasmid (EAF) that encodes both the BFP fimbria associated with bacterial adherence to the intestine cells and the *perABC* operon whose products control the transcription of the chromosomal LEE region (Pearson et al., 2016; Serapio-Palacios and Finlay, 2020). Deletion of *relA* partially impaired EPEC adherence to epithelial cells by diminishing the transcription of the *perABC* operon that controls the expression of the adhesins BFP and intimin (Spira et al., 2014). However, gratuitous overproduction of (p)ppGpp slightly inhibited the expression of *perABC*. The antagonistic effects of (p)ppGpp on *perABC* expression suggests that a fine-tuned concentration of (p)ppGpp is required to maximize EPEC adherence. Even though (p)ppGpp concentrations were not assessed in different EHEC and EPEC isolates the data presented in these studies suggest that the expression of virulence genes and virulence traits are modulated by this alarmone.

Shiga toxin-producing *E. coli* (STEC) is another diarrheogenic pathotype that secretes Shiga toxin, but unlike EHEC, does not harbors a LEE and, consequently, does not display intimate adherence to intestinal cells (Bryan et al., 2015; Joseph et al., 2020). The role of (p)ppGpp in STEC virulence and particularly in toxin production and secretion has been examined in detail. The *stx* genes that encode Shiga toxin were introduced in the STEC genome by means of lambdoid bacteriophages, a phenomenon known as phage lysogenic conversion (Harrison and Brockhurst, 2017). The synthesis and release of Shiga toxin is preceded by the induction of the bacteriophage, a development that eventually results in cell lysis (Waldor and Friedman, 2005; Nowicki et al., 2013). Therefore, the level of Shiga-toxin production and release is directly related to the number of STEC bacteria in a population undergoing phage induction. On the other hand, (p)ppGpp has been shown to inhibit stx phage replication, as the Δ*relA* Δ*spoT* double mutant displayed a higher degree of phage DNA replication and formed larger plaques on Δ*relA* Δ*spoT* lawns (Nowicki et al., 2013). A subsequent report has shown that intrinsic (p)ppGpp concentration is indeed inversely correlated with Stx toxin production, as STEC strains showing higher cytotoxicity toward Vero cells (the golden standard method for measuring toxin production and STEC virulence) usually contained lower levels of (p)ppGpp (Stella et al., 2017).

The extraintestinal uropathogenic *E. coli* (UPEC) causes recurrent infections in the urinary tract. A critical mechanism of UPEC infection is the ability to invade the bladder cells by means of Type-I fimbriae. The expression of fimbrial genes is controlled by (p)ppGpp and DksA (Aberg et al., 2008). DksA is a transcription factor that binds to RNA polymerase and greatly enhances the effect of (p)ppGpp on transcription regulation (Gourse et al., 2018). (p)ppGpp activates the promoter of *fimB* that encodes a recombinase that specifically inverts the promoter of the *fimAICDFGH* operon. This operon codes for the structural components of the type-I fimbria. By inverting the promoter orientation FimB allows the transcription of the *fimAICDFGH* operon switching the promoter from “off” to “on” state (Eisenstein, 1981). Amino acid starvation or growth arrest caused by bacteria entering the stationary phase increase (p)ppGpp which activates the *fimB* and *fimA* promoters (Aberg et al., 2006). Likewise, *relA* overexpression also induces the transcription from these promoters resulting in the synthesis of Type-I fimbria and the invasion and colonization of bladder cells. Altogether, the data suggest that UPEC strains with high (p)ppGpp intrinsic levels present higher levels of virulence toward the host.

Lastly, (p)ppGpp is directly associated with the pathogenicity of many bacterial species and is required for the full expression of virulence genes (Dalebroux et al., 2010; Kalia et al., 2013). Interestingly, STEC, the only *E. coli* pathotype in which a populational study correlating (p)ppGpp and pathogenicity has been performed stands out as an outlier. STEC toxin production is coupled to phage induction, which is inhibited by (p)ppGpp. By inhibiting phage replication (p)ppGpp acts as a legitimate promoter of bacterial survival.

## Conclusions

The intrinsic concentration of (p)ppGpp in strains of the species *Escherichia coli* is not constant. Rather, the level of (p)ppGpp is been shaped by the historical contingency of bacterial populations. There are two types of evidence that support this assertion: direct assessment of (p)ppGpp in *E. coli* natural isolates and the selection of *relA* and *spoT* mutant alleles in evolution experiments. These data indicate that the genes that govern (p)ppGpp synthesis and degradation are subjected to frequent microevolutionary pressures that will eventually determine the optimal concentration of (p)ppGpp in a population. Given the pleiotropic effects of (p)ppGpp in the cell, adjustments of (p)ppGpp intrinsic concentration should have broad implications on bacterial physiology (Figure 3). In fact, intrinsic variations in (p)ppGpp levels differentially affect growth, stress response, virulence and antibiotic resistance. However, the intrinsic levels of (p)ppGpp in *E. coli* natural isolates do not perfectly correlate with the expected phenotypes. For instance, growth rate and (p)ppGpp inverse correlation across the ECOR strains was significant but not perfect, which suggests that the role of this alarmone in growth is intertwined with other regulatory circuits and that bacterial physiology is always more complex than firstly assumed.

## Author Contributions

BS and KO drafted the manuscript. All authors contributed to the article and approved the submitted version.

## Conflict of Interest

The authors declare that the research was conducted in the absence of any commercial or financial relationships that could be construed as a potential conflict of interest.
